# HDAC6 is associated with the formation of aortic dissection in human

**DOI:** 10.1186/s10020-019-0080-7

**Published:** 2019-03-29

**Authors:** Xian Guo, Ze-Min Fang, Xiang Wei, Bo Huo, Xin Yi, Cai Cheng, Jun Chen, Xue-Hai Zhu, Anas Omar Khalil Abu Bokha, Ding-Sheng Jiang

**Affiliations:** 1 Division of Cardiothoracic and Vascular Surgery, Tongji Hospital, Tongji Medical College, Huazhong University of Science and Technology, Wuhan, 430030 China; 2 Key Laboratory of Organ Transplantation, Ministry of Education, Wuhan, China; 3 NHC Key Laboratory of Organ Transplantation, Wuhan, China; 4 Key Laboratory of Organ Transplantation, Chinese Academy of Medical Sciences, Wuhan, China; 5 Department of Cardiology, Renmin Hospital of Wuhan University, Wuhan, 430060 China; 6 Tongji Medical College, Huazhong University of Science and Technology, Wuhan, 430030 China

**Keywords:** Aortic dissection, Histone post-translational modification, HDAC6, H3K23ac, H4K20me2, Signaling pathway

## Abstract

**Background:**

The pathological features of aortic dissection (AD) include vascular smooth muscle cell (VSMC) loss, elastic fiber fraction, and inflammatory responses in the aorta. However, little is known about the post-translational modification mechanisms responsible for these biological processes.

**Methods:**

A total of 72 aorta samples, used for protein detection, were collected from 36 coronary artery disease (CAD, served as the control) patients and 36 type A AD (TAAD) patients. Chromatin immunoprecipitation (ChIP)-PCR was used to identify the genes regulated by H3K23ac, and tubastatin A, an inhibitor of HDAC6, was utilized to clarify the downstream mechanisms regulated by HDAC6.

**Results:**

We found that the protein level of histone deacetylase HDAC6 was reduced in the aortas of patients suffering from TAAD and that the protein levels of H4K12ac, and H3K23ac significantly increased, while H3K18ac, H4K8ac, and H4K5ac dramatically decreased when compared with CAD patients. Although H3K23ac, H3K18ac, and H4K8ac increased in the human VSMCs after treatment with the HDAC6 inhibitor tubastatin A, only H3K23ac showed the same results in human tissues. Notably, the results of ChIP-PCR demonstrated that H3K23ac was enriched in extracellular matrix (ECM)-related genes, including Col1A2, Col3A1, CTGF, POSTN, MMP2, TIMP2, and ACTA2, in the aortic samples of TAAD patients. In addition, our results showed that HDAC6 regulates H4K20me2 and p-MEK1/2 in the pathological process of TAAD.

**Conclusions:**

These results indicate that HDAC6 is involved in human TAAD formation by regulating H3K23ac, H4K20me2 and p-MEK1/2, thus, providing a strategy for the treatment of TAAD by targeting protein post-translational modifications (PTMs), chiefly histone PTMs.

**Electronic supplementary material:**

The online version of this article (10.1186/s10020-019-0080-7) contains supplementary material, which is available to authorized users.

## Background

Aortic dissection (AD) is associated with significant morbidity and mortality, and is a life-threatening aortic disease (Isselbacher et al., 2016). Because no conservative pharmacological approach can effectively prevent the progression or the risk of aortic rupture, surgical repair remains the only current treatment option for AD (Maegdefessel et al., 2014). This situation likely stems from the fact that the pathophysiological mechanisms of AD are not fully understood. Numerous studies have indicated that the aorta of AD patients undergoes pathological changes that include medial degeneration (e.g., smooth muscle cell loss, elastin fragmentation and degeneration), extracellular matrix (ECM) degradation, and inflammatory cell infiltration (Maegdefessel et al., 2014; Erbel et al., 2014). Nevertheless, the mechanisms that regulate these pathological processes of the aorta to control AD formation remain largely unclear.

Previous studies demonstrated that histone post-translational modifications (PTMs), especially histone acetylation, play critical roles in vascular smooth muscle cells (VSMCs) and vascular homeostasis (Chang et al., 2006). Increasing evidence has shown that the proliferation of VSMCs and endothelial cells, vascular remodeling and inflammation, which are closely related to AD occurrence, are regulated by histone acetylation modifiers (Pons et al., 2009). For example, *Olson EN* and his colleagues demonstrated that endothelial cell proliferation and migration were affected by histone deacetylase (HDAC) 7 (Wang et al., 2008), and HDAC7 maintains vascular integrity by suppressing matrix metalloproteinase 10 expression (Chang et al., 2006). HDAC4 regulated proliferation and migration of VSMCs via activation of p38 mitogen-activated protein kinase/heat shock protein 27 signals (Usui et al., 2014). Additionally, MCT-1, an HDAC inhibitor, reduced angiotensin II-induced abdominal aortic aneurysm formation in ApoE mice (Vinh et al., 2008). However, the expression pattern of HDACs in patients with AD is still unknown. Furthermore, the mechanisms that mediate the function of HDACs during AD formation require further investigation. Thus, in this study, we investigated the expression patterns of HDACs, and how HDACs regulate histone acetylation, methylation and related signaling pathways in the aortas of TAAD and coronary artery disease (CAD) patients to elucidate the role of histone PTMs in human TAAD formation.

Our results demonstrated that H3K23 acetylation (H3K23ac), H4K12ac, H3K23 mono-methylation (H3K23me1) and H3K9me1 levels significantly increased, while H4K20me2, H3K18ac, H4K5ac, and H4K8ac levels were remarkably reduced in the aorta of type A AD (TAAD) patients compared with CAD patients. Furthermore, we demonstrated that histone deacetylase (HDAC) 6 protein levels were significantly decreased in patients with TAAD and that HDAC6 could regulate ECM secretion via deacetylating H3K23. Additionally, we found that H4K20me2 and p-MEK1/2 function downstream of HDAC6 to mediate the potential effects of HDAC6 on TAAD.

## Methods

### Human aorta samples

In this study, we included 72 cases of aortic samples collected from 36 patients with TAAD and 36 patients with CAD (served as the control). Each aortic tissue was weighed to be the same, and 6 samples were grouped randomly as a pool for the following experiments. For this investigation, the dissected segment was removed from patients with TAAD when they underwent surgical treatment. For patients who underwent coronary artery bypass surgery (CABG), a hole punched from the normal aorta that was used for the bypass graft connection was collected for the study. Once the tissue samples were removed, they were cut into pieces equivalent to the size of soybeans with sterile instruments, packed into cryogenic vials, stored in ice temporarily, and then transferred into liquid nitrogen as soon as possible. Finally, specimens were preserved in a cryogenic refrigerator at − 80 °C. Patients with iatrogenic or traumatic TAAD, hereditary disease (e.g., Marfan syndrome), cancers, or autoimmune disease were excluded from this study. The patients’ clinical information can be found in Table 1. Informed consent about the storage and possibility of scientific research usage of intraoperative tissue specimens was obtained from all subjects before surgery from both the TAAD and CAD groups. This study was approved by the Tongji Hospital, Tongji Medical College, Huazhong University of Science and Technology Review Board in Wuhan, China.

**Table 1:** The clinic information of patients

Clinical Indicators	CAD	TAAD
Sex (Male/Female)	27/9	27/9
Age (year)	60.3 ± 5.1	53.8 ± 8.0^**^
BMI (kg/m^2^)	23.4 ± 2.9	23.4 ± 1.9
Smokers (*n*, %)	18 (50.0%)	19 (52.7%)
Diabetes (*n*, %)	14 (38.9%)	0 (0.0%)^**^
Hypertension (*n*, %)	24 (66.7%)	25 (69.4%)
NYHA classification	2.4 ± 1.0	2.2 ± 0.8
SBP (mmHg)	125.7 ± 17.7	134.0 ± 29.8
Aortic Diameter (mm)	32.2 ± 3.3	41.6 ± 7.7^**^
EF (%)	53.9 ± 14.1	60.2 ± 4.2^*^
WBC (10^9^/L)	7.6 ± 3.9	11.5 ± 4.3^**^
hs-TnI (μg/L)	21.4 (10.0, 171.0)	66.8 (9.6, 528.8)
pro-BNP (pg/mL)	677.5 (222, 2285)	450 (274, 891)
D-dimer (μg/L)	NA	22.1 ± 13.3

*CAD* Coronary artery disease, *TAAD* Type A aortic dissection, *BMI* Body mass index, *SBP* Systolic blood pressure, *NYHA classification* New York Heart Association classification, *EF* Ejection fraction, *WBC* White blood cell. Aortic diameter means the diameter of ascending aorta except when patients with aneurysm of descending aorta that means the diameter of descending aorta, *NA* Not available. *Indicates significant changes from TAAD compared with CAD, *p < 0.05; **p < 0.01

### Aortic diameter measurement

Aortic diameters were measured as the peak of the ascending aorta in TAAD patients by images of CT angiography. For CAD subjects, aortic diameters were measured and reported by a sonographer when transthoracic echocardiography (TTE) was performed to routinely assess each patient’s cardiac function.

### Western blot and antibody information

Western blot analyses were performed as previously described (Jiang et al., 2016; Jiang et al., 2017a; Jiang et al., 2017b). The HDAC Antibody Sampler Kit (#9928), Phospho-HDAC4^Ser246^/HDAC5^Ser259^/HDAC7^Ser155^ (#3443), HDAC2 (#5113), HDAC6 (#7558), H3K4me2 (#9725), H3K4me3 (#9727), H3K27me2 (#9728), H3K27me3 (#9733), Phospho-AKT^Ser473^ (#4060), AKT (#4685), Phospho-mTOR^Ser2448^ (#5536), mTOR (#2983), Phospho-AMPKα^Thr172^ (#2535), AMPKα (#5831), Phospho-MEK1/2^Ser217/221^ (#9154), Phospho-ERK1/2^Thr202/Tyr204^ (#4370), ERK1/2 (#4695), Phospho-JNK1/2 ^Thr183/Tyr185^ (#4668), JNK1/2 (#9258), Phospho-p38^Thr180/Tyr182^ (#4511), p38 (#8690), Phospho-β-Catenin^Ser675^ (#4176), Non-phospho-β-Catenin^Ser33/37/Thr41^ (#8814), β-Catenin (#8480), Phospho-IKKα/β^Ser176/180^ (#2697), IKKβ (#8943), Phospho-NF-κB p65^Ser536^ (#3033), NF-κB p65 (#8242), Phospho-Smad2^Ser465/467^ (#3108), Smad2 (#5339), Phospho-Smad3^Ser423/425^ (#9520), Smad3 (#9523), Phospho-Smad1/5^Ser463/465^ (#9516), and Smad1 (#6944) antibodies were obtained from Cell Signaling Technology. Antibodies against Histone H3 (ab1791), H3K36me2 (ab9049), H3K36me3 (ab9050), H4K20me3 (ab9053), H3K9me1 (ab9045), H3K9me2 (ab1220), H3K4me1 (ab8895), H3K23me1 (ab176132), H4K5ac (ab51997), H4K8ac (ab15823), H3K18ac (ab40888), H3K23ac (ab61234) and H4K12ac (ab46983) were purchased from Abcam.

### Isolation of histone proteins

Histone proteins were extracted from aortic tissues based on the following protocol. In brief, aortal tissues were homogenized by using a Dounce homogenizer in 1 mL of hypotonic lysis buffer (10 mM Tris-HCl (pH 8.0), 1 mM KCl, 1.5 mM MgCl_2_, 1 mM DTT, 0.1 mM EGTA, 1 mM EDTA, Phos-STOP, and Complete protease inhibitor cocktail). Centrifugation was performed at 5000×g for 10 min at 4 °C after incubating homogenized tissues on ice for 10 min. The pellet was resuspended in 1 mL of hypotonic lysis buffer, and NP-40 was added to a final concentration of 0.1%. The mixture was then shaken for 60 min on a Roto-Shake. The samples were centrifuged at 10000×g for 10 min at 4 °C, and the pellet was saved and resuspended in 1 mL of 0.2 M H_2_SO_4_ with gentle pipetting. After shaking in a Roto-Shake Genie for 3 h at 4 °C, the samples were centrifuged at 12500×g for 10 min at 4 °C, and then, the supernatant was transferred to a new tube. Next, 100% trichloroacetic acid was added to a final concentration of 10% and incubated on ice for 30 min to precipitate the histones. The centrifugation was performed at 12500×g for 10 min at 4 °C, and the pellets were saved and washed with 1 mL of 100% ice-cold acetone and a final concentration of 0.1% HCl 3 times. After drying with a vacuum centrifuge, histones were dissolved in ddH_2_O and the concentration was quantitated using a BCA kit (Thermo Fisher Scientific, 23227).

### Cell culture and treatment

The rabbit aortic vascular smooth muscle cells (RAVSMCs) were cultured with DMEM/F12 (SH30023.01; HyClone) supplemented with 10% fetal bovine serum (FBS; 1767839; Thermo Fisher Scientific), and 1% penicillin-streptomycin (15140–122; Thermo Fisher Scientific).

Human normal aorta tissues used for primary culture VSMCs (HAVSMCs) were obtained from recipients who underwent heart transplantation at Tongji Hospital, Tongji Medical College, Huazhong University of Science and Technology. Aortic tissues were divided into pieces of approximately 1.5mm^2^ and stored in DMEM/F12 with 1% penicillin-streptomycin at 4 °C. Then, tissues were transferred into a new petri dish after removal of blood stains and connective tissue. The intima and media structures were identified under a stereo microscope and stripped of the intimal and residual adventitial tissues with forceps. The dissected media of the vessels were then cut into small pieces (1–2 mm) and transferred to cell culture flasks. The tissue blocks were spread evenly on the bottom of the flask with a control interval of approximately 2 mm. Five milliliters of DMEM/F12 medium supplemented with 10% FBS, 1% L-glutamine, and antibiotics was added to the flask, and the lid was loosely screwed on. The flask was placed in the incubator and stood upright for 30 min to allow explant attachment to the wall of the culture flask. After 30 min, the culture bottle was then lowered. The culture bottle was not moved for 5 days. A long spindle-shaped smooth muscle cell was observed around the tissue block in approximately one week. After the cells grew, the medium was renewed every 3 days, and the state of the cells was closely observed. The smooth muscle cells around the tissue block were evenly distributed, and the cells were routinely passaged when the degree of cell confluence was approximately 80%. After starvation for 12 h, RAVSMCs and HAVSMCs were treated with tubastatin A (S8049, Selleck) at different concentrations (0, 1, 5, 10, 15, 20 μM) for 48 h.

### Chromatin immunoprecipitation (ChIP)-PCR

Approximately 70 mg of frozen aortic tissues per sample was used to isolate genomic DNA. Ground tissues were resuspended in DMEM culture medium and fixed with 1.5% formaldehyde for 15 min, and then, 0.125 M glycine was added. The medium was removed, and the tissues were washed with cold PBS twice, followed by homogenization in a Dounce homogenizer in 1 mL of PBS. The cell pellets were harvested, and 1 mL cell collection buffer (100 mM Tris-HCl pH = 9.4, 10 mM DTT, with Complete protease inhibitor cocktail, Roche) was added. Cells were lysed on ice for 20 min, and the second homogenization was conducted. The deposits were collected and washed twice with PBS. Cell pellets were pretreated with Nucleus/Chromatin Preparation buffer (NCP buffer I: 10 mM EDTA, 0.5 mM EGTA, 10 mM HEPES pH = 6.5, 0.25% Triton X-100; Buffer II: 1 mM EDTA, 0.5 mM EGTA, 10 mM HEPES pH = 6.5, 200 mM NaCl) and then lysed with nuclear lysis buffer (10 mM EDTA, 50 mM Tris-HCl pH = 8.1, 1% SDS, Complete protease inhibitor cocktail) on ice for 15 min. Sonication was performed to generate a DNA fragment of 400–800 bp, with a No.2 microtip at a power output of 25% (on 6 s, off 30 s, 10 times). Chromatin quantified to 100 μg DNA was utilized for further immunoprecipitation and mixed with IP buffer (2 mM EDTA, 150 mM NaCl, 20 mM Tris-HCl pH = 8.1, 0.1% Triton X-100, Complete protease inhibitor cocktail) in a total volume of 1 mL. The chromatin was diluted at a rate of 1:10 in IP buffer as the input. Protein A/G magnetic beads (Biotool, B23202) were washed twice with TE buffer (10 mM Tris-HCl pH = 8.1, 1 mM EDTA). Then, the chromatin was precleared for IP with the prepared beads in a volume of 30 mL and rotated at 4 °C for 3 h. The supernatants were transferred to fresh microcentrifuge tubes, and 2 μg of H3K23ac antibody (Abcam, ab61234) or IgG (Santa Cruz, sc2025) was added to each sample and rotated overnight at 4 °C. The next day, 40 μL protein A/G magnetic beads were added into each antibody-chromatin sample, and rotation was performed for 2 h at 4 °C. Beads were harvested with a magnetic holder and washed in the shaker sequentially for 5 min at 4 °C in 1 mL of wash buffer I (2 mM EDTA, 20 mM Tris-HCl pH = 8.1, 0.1% SDS, 1% Triton X-100, 150 mM NaCl) × 2, wash buffer II (2 mM EDTA, 20 mM Tris-HCl pH = 8.1, 0.1% SDS, 1% Triton X-100, 500 mM NaCl), and TE buffer× 2. Beads were resuspended in 250 μL extraction buffer (1% SDS, 0.1 M NaHCO_3_), and rotated at room temperature for 30 min, the supernatants were collected, and this step was repeated one more time. The confluent supernatants were chromatin eluted, following the addition of 10 μg RNase A (Sigma-Aldrich, R5125) for RNA digestion at 37 °C for 2 h. All samples, including IP and input samples were mixed with 120 μg proteinase K to reverse formaldehyde crosslinks at 65 °C overnight. DNA was purified with a PCR purification kit (Beyotime, D0033), and the chromatin was dissolved in 120 μL TE buffer. Then, 2 μL of DNA solution per well was loaded for the real-time PCR assay. The primers used in this study are listed in Additional file 1: Table S1.

### Data analysis

All data are expressed as the mean ± SD (standard deviation). For the TAAD and CAD comparisons, Student’s t-test was used to analyze all the data by using SPSS software (version 13.0). The difference was considered statistically significant when the *p*-value was less than 0.05.

## Results

### HDAC6 deacetylates H3K23 to participate in TAAD formation

**Fig. 1:**
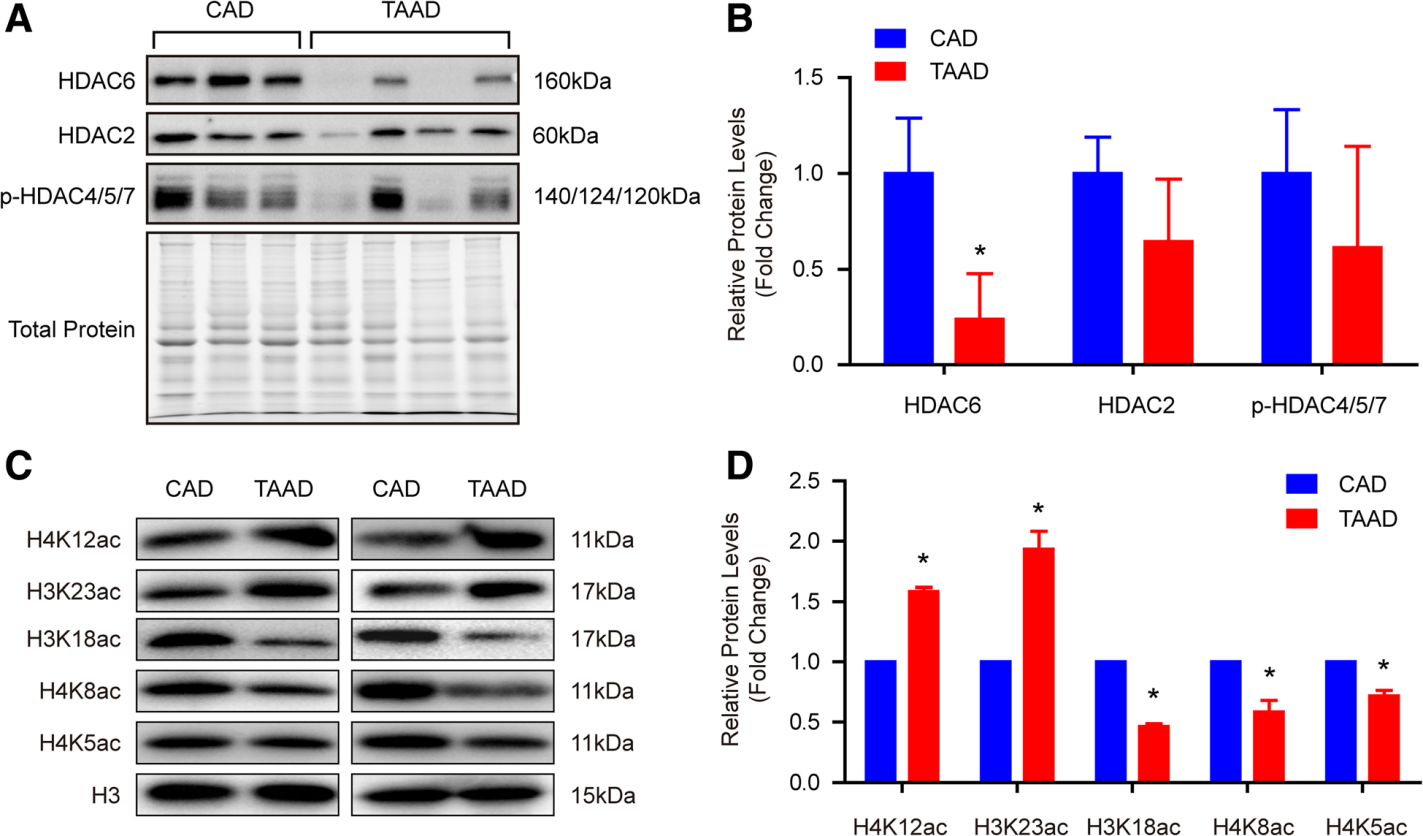
HDAC6 protein level was decreased in aorta of TAAD patients. **a** and **b** The protein levels of histone deacetylases were detected by western blot (*n* = 6 pooled samples per group), *p < 0.05 vs. CAD. **c** and **d** The acetylation levels of H4K12, H3K23, H3K18, H4K8, H4K5 were evaluated by western blot in the aorta of CAD and TAAD patients (*n* = 6 pooled samples per group), *p < 0.05 vs. CAD

**Fig. 2:**
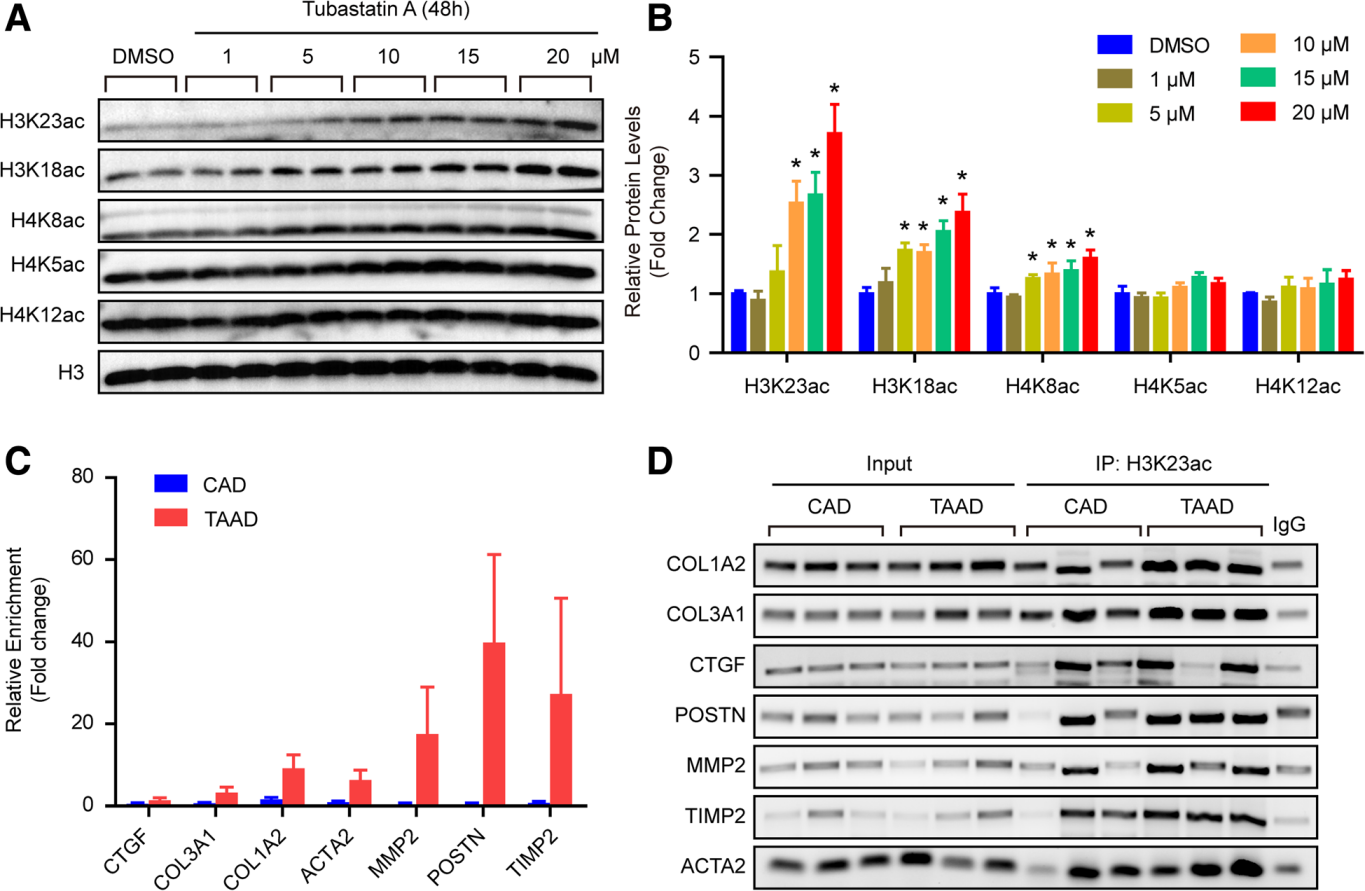
HDAC6 regulates ECM secretion via H3K23ac. **a** and **b** Histone acetylation, including H3K23, H3K18, H4K8, H4K5, and H4K12, were verified in rabbit aortic VSMCs treated with DMSO or different concentrations of tubastatin A (*n* = 4 samples per group), *p < 0.05 vs. DMSO. **c** The statistical result of ChIP-PCR. **d** The ChIP-PCR products were separated by agarose gel electrophoresis (*n* = 6 pooled samples per group)

To address the role of histone acetylation in the pathology of TAAD, we first collected 36 aortic tissues from TAAD patients and CAD patients (as normal controls) in each group. Detailed clinical information is provided in Table 1. Compared with CAD patients, TAAD patients had an enlarged aortic diameter but a younger age and a low incidence of diabetes. The protein levels of HDAC families were detected in the aortas of CAD and TAAD patients using an HDAC Antibody Sampler Kit from Cell Signaling Technology. As shown in Fig. 1a and b, HDAC6 protein levels sharply decreased in the TAAD patients, but a comparable level of HDAC2 and p-HDAC4/5/7 was observed between CAD and TAAD patients. The HDAC family comprises histone deacetylases that deacetylate histone proteins. As HDAC6 protein levels were reduced in TAAD patients, we investigated which histone sites were deacetylated by HDAC6 during TAAD formation. The western blot results demonstrated that H4K12ac and H3K23ac levels were significantly elevated, while H3K18ac, H4K8ac, and H4K5ac levels were decreased remarkably in TAAD patients compared with CAD patients (Fig. 1c and d). These results indicated that H3K23ac and H4K12ac are potential targets of the decreased deacetylase HDAC6 in the aortas of TAAD patients. To further determine the target of HDAC6, we treated RAVSMCs with different concentrations of the HDAC6-specific inhibitor tubastatin A. Tubastatin A treatment significantly increased H3K23ac, H3K18ac, and H4K8ac levels at concentrations as low as 10 μM in RAVSMCs, while comparable levels of H4K5ac and H4K12ac were detected (Fig. 2a and b). Because tubastatin A did not regulate H4K12ac, the in vivo and in vitro results suggested that H3K23ac is the target of HDAC6 during VSMC dysfunction and TAAD occurrence. Next, we investigated whether or not increased H3K23ac levels influence ECM secretion in the human aorta. ChIP-PCR was utilized to identify and quantify the binding of H3K23ac to ECM related genes. As shown in Fig. 2c and d, more H3K23ac enrichment at the CTGF, COL3A1, COL1A2, POSTN, ACTA2, MMP2, and TIMP2 genes were observed in the aortas of TAAD patients compared with CAD patients. The results suggested that HDAC6 may play an essential role in ECM homeostasis via regulating H3K23 deacetylation to affect TAAD occurrence.

### H4K20me2 was regulated by HDAC6 during TAAD formation

**Fig. 3:**
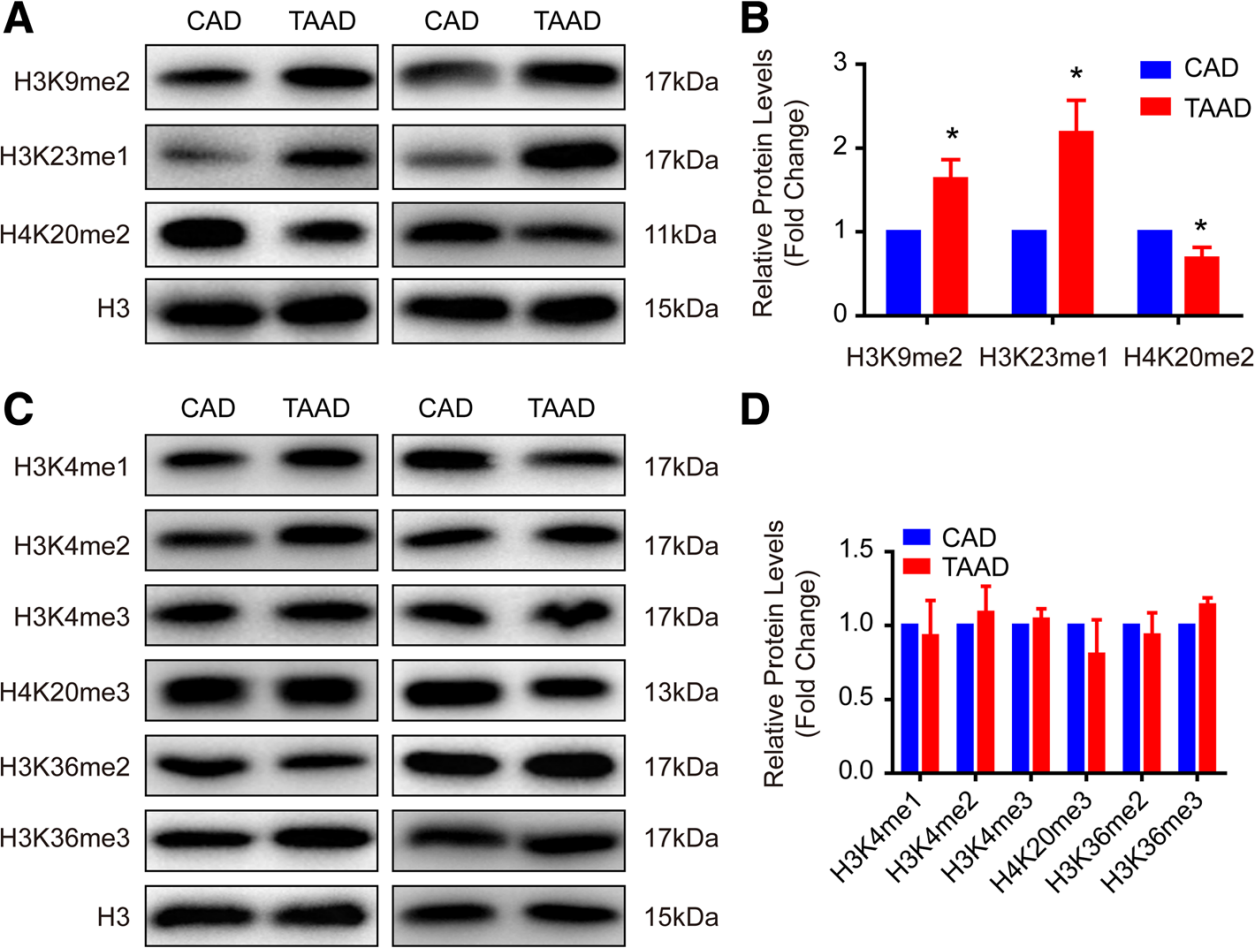
Histone methylation in samples of human aortic wall. **a**-**d** The protein levels of histone methylation were verified by western blotting in the aorta samples of CAD and TAAD patients, (**a** and **b**) differentially expressed histone methylation in TAAD patients; (**c**-**d**) comparable histone methylation in two groups. *n* = 6 pooled samples per group, *p < 0.05 vs. CAD

**Fig. 4:**
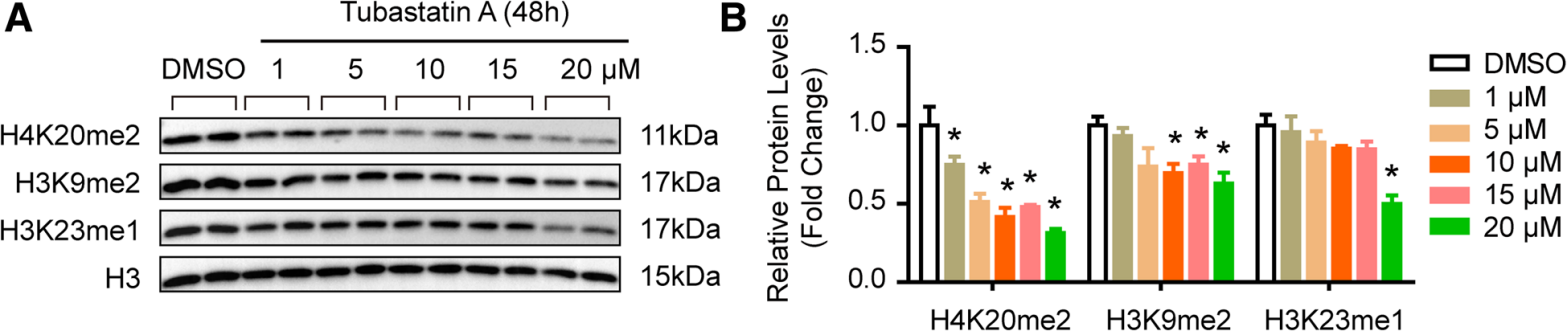
Histone methylation regulated by HDAC6. **a** and **b** The protein levels of H4K20me2, H3K9me2, and H3K23me1 were detected by western blot in primary cultured HAVSMCs treated with DMSO or different concentrations of tubastatin A (*n* = 4 samples per group), *p < 0.05 vs. DMSO

Since histone acetylation and methylation always work together to regulate gene expression and disease, the methylation of histone proteins was detected in the aortas of CAD and TAAD patients. H3K9me2 and H3K23me1 increased in TAAD patients, but H4K20me2 decreased compared with CAD patients (Fig. 3a and b). However, comparable levels of H3K4me1, H3K4me2, H3K4me3, H4K20me3, H3K36me2, and H3K36me3 were observed between CAD and TAAD patients (Fig. 3c and d). To further investigate whether HDAC6 regulates H4K20me2, H4K9me2, and H3K23me1 in VSMCs, we treated primary cultured HAVSMCs with the HDAC6 inhibitor tubastatin A at different concentrations. Tubastatin A remarkably inhibited H4K20me2 at concentrations as low as 1 μM, and H4K9me2 and H3K23me1 were suppressed at 10 or 20 μM respectively (Fig. 4a and b). These results indicated that reduced HDAC6 in the aortas of TAAD patients results in decreased H4K20me2 levels.

### HDAC6 cooperates with the MEK signaling pathway to participate in TAAD pathology

**Fig. 5:**
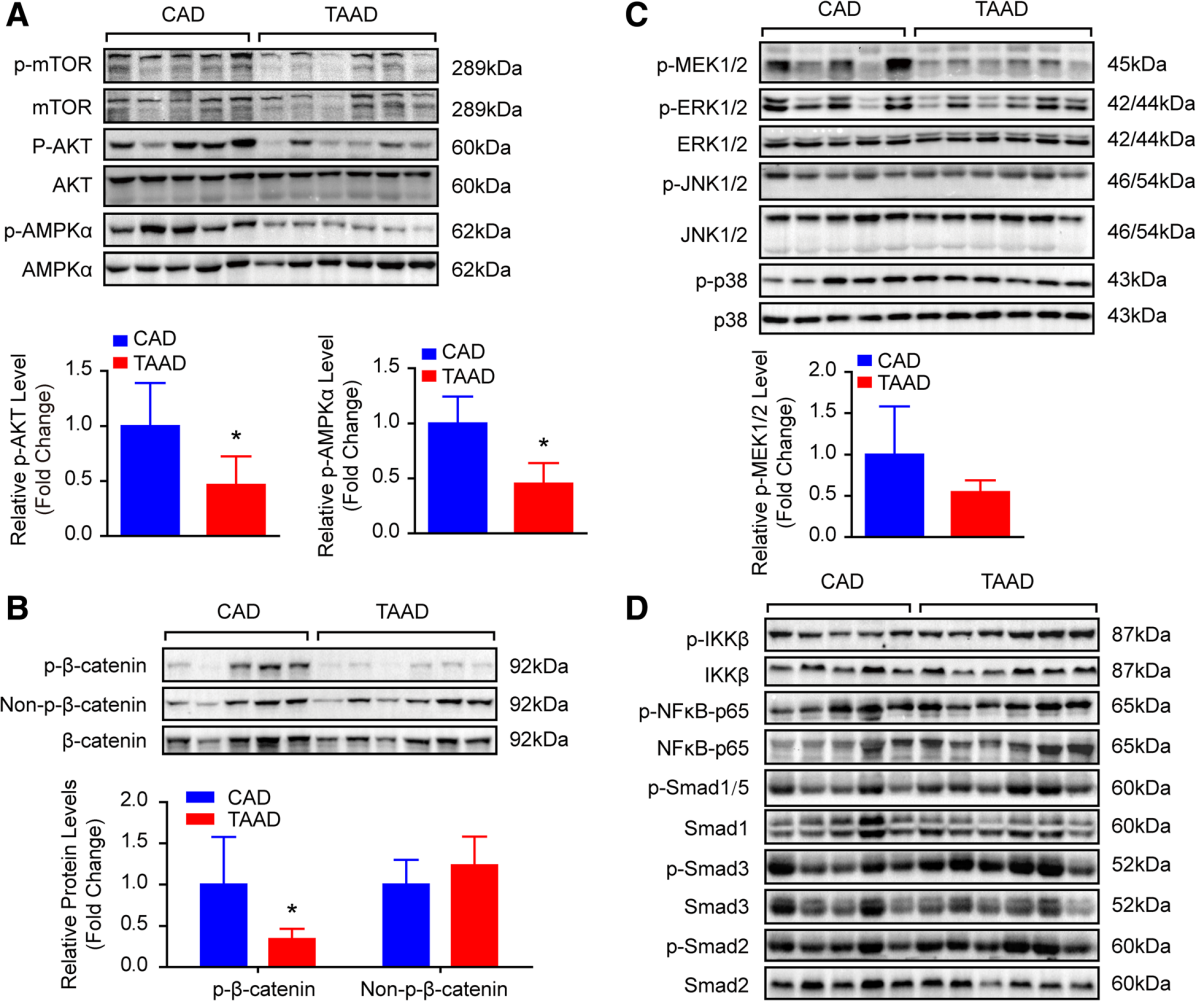
The signaling pathways evaluated in human aorta samples. The phosphorylation and total protein levels of key molecules in signaling pathways were detected by western blot in the aorta of CAD and TAAD patients. **a** The phosphorylation and total protein levels of mTOR, AKT and AMPKα; (**b**) The phosphorylation, non-phosphorylation and total protein levels of β-catenin; (**c**) The phosphorylation and total protein levels of MEK1/2, ERK1/2, JNK1/2, and P38; (**d**) The phosphorylation and total protein levels of IKKβ, NF-κB-p65, Smad1/5, Smad2, and Smad3. *n* = 6 pooled samples per group, *p < 0.05 vs. CAD

VSMC apoptosis, ECM abnormalities, and inflammatory infiltration are typical characteristics in the aortas of TAAD patients (Li et al., 2018), and many signaling pathways, including AKT, AMPK, MAPK, β-catenin, NF-κB, and Smad pathways, are closely related to these biological processes. We investigated whether these signaling pathways contribute to TAAD formation. First, we detected key molecules in these pathways in the aortas of TAAD patients. Our results showed that compared with CAD patients, the phosphorylation levels of AKT^Ser473^, AMPKα^Thr172^, and β-catenin^ser675^ were sharply decreased in the aorta of TAAD patients, while p-mTOR, and non-p-β-catenin maintained similar levels between the two groups (Fig. 5a and b). In the MAPK signaling pathway, phosphorylation levels of MEK1/2 were reduced without significant differences in TAAD patients, and comparable p-ERK1/2, p-JNK1/2, and p-P38 levels were observed in the aortas of CAD and TAAD individuals (Fig. 5c). However, the NF-κB and Smad signaling pathways may be independent of TAAD formation, as evidenced by the parallel phosphorylation levels of IKKβ, NF-κB-p65, Smad1/5, Smad2, and Smad3 between CAD and TAAD patients (Fig. 5d). These results demonstrated that AKT, AMPKα, β-catenin, and MEK1/2 signaling pathways might contribute to TAAD pathological processes.

**Fig. 6:**
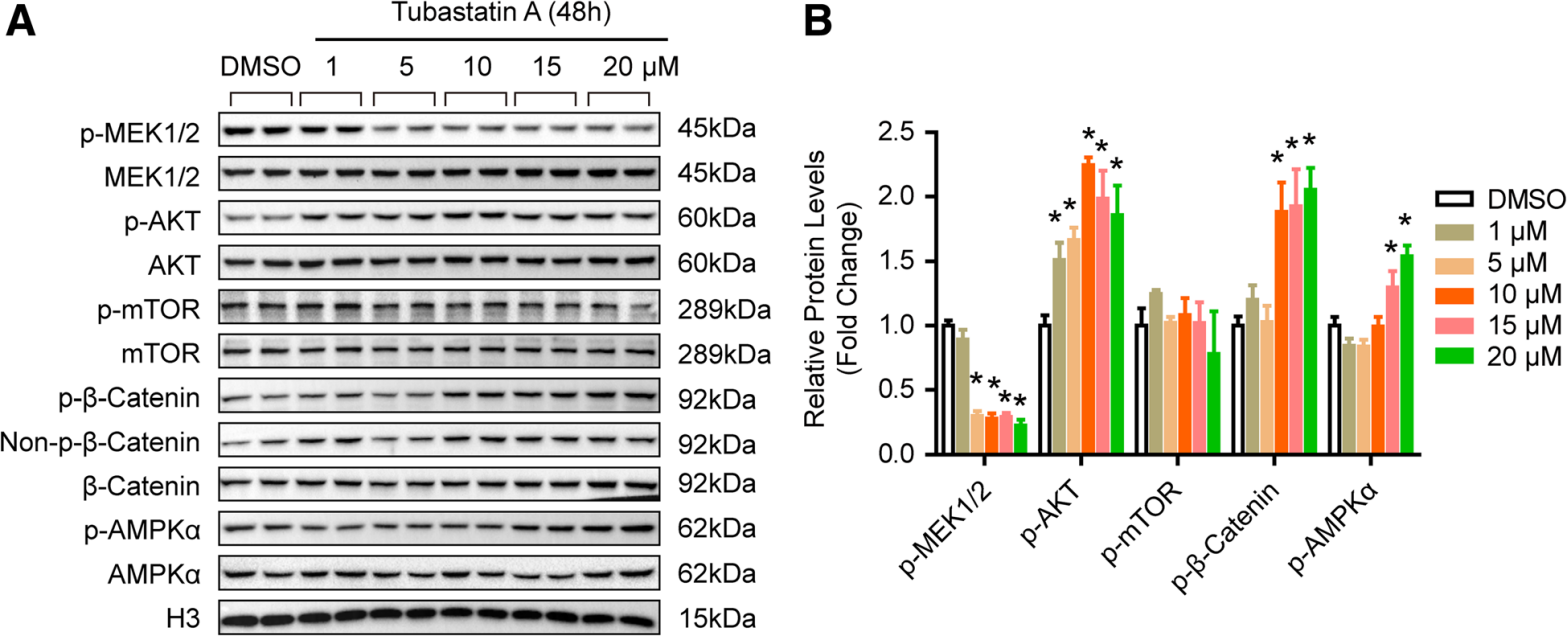
The signaling pathways regulated by HDAC6. **a** and **b** Western blot was used to detect the signaling molecules in the primary cultured HAVSMCs treated with DMSO or different concentrations of tubastatin A (*n* = 4 samples per group), *p < 0.05 vs. DMSO

Similarly, we treated primary cultured HAVSMCs with tubastatin A to investigate which signaling pathways were regulated by HDAC6 during TAAD formation. As shown in Fig. 6a and b, the phosphorylation levels of AKT, β-catenin, and AMPKα were significantly increased after tubastatin A treatment. However, MEK1/2 activation was inhibited by tubastatin A (Fig. 6a and b). In addition, the mTOR signaling pathway was not affected by tubastatin A (Fig. 6a and b). Taken together, these results indicate that the HDAC6-MEK pathway participates in the regulation of pathological progress in TAAD.

## Discussion

In this work, we found that the histone deacetylase HDAC6 was substantially reduced in the aortas of TAAD patients and that HDAC6 regulates ECM secretion by deacetylating H3K23. In addition, our results showed that p-MEK1/2 and H4K20me2 were regulated by HDAC6 to affect TAAD. Overall, these new findings could provide potential therapeutic targets and unprecedented insights into the pathogenesis of TAAD.

In addition to VSMC loss/phenotype switching, ECM degradation and inflammatory infiltration were involved in the pathogenesis of AD (Erbel et al., 2014; Li et al., 2018). An increasing number of studies have indicated that these pathological processes are closely related to epigenetic mechanisms, especially histone modifications. Previous studies demonstrated that HDAC4 controls PDGF-BB-induced VSMC proliferation and migration to affect neointimal hyperplasia (Usui et al., 2014). Myocyte enhancer factor-2 (MEF2) recruits HDAC7 to the promoter of MMP10 to suppress its expression, which maintains vascular integrity by protecting the ECM from degradation (Chang et al., 2006). Since HDACs contribute to VSMC function and ECM secretion, we examined HDAC protein levels in the aorta of humans. Our results showed that the HDAC6 expression level was notably decreased in TAAD patients compared with the CAD counterparts. In the pulmonary artery smooth muscle cells (PASMCs) of patients with pulmonary arterial hypertension (PAH), HDAC6 expression level was significantly upregulated, and inhibition of HDAC6 could reduce PASMC proliferation and resistance to apoptosis and finally improve established PAH (Boucherat et al., 2017). Co-culturing VSMCs with endothelial cells (ECs) could increase EC migration, which is linked with elevated HDAC6 expression and low levels of acetylated tubulin in ECs. Furthermore, knockdown of HDAC6 by siRNA could reverse the migration of ECs that resulted from coculture with VSMCs (Wang et al., 2010). Wu et al. reported that inhibition of HDAC6 by tubastatin A suppressed PDGF-BB induced VSMC proliferation and migration in vitro, and decreased neointimal hyperplasia in response to injury in vivo (Wu et al., 2016). These studies indicate that HDAC6 plays a critical role in VSMCs and ECs. Given that reduced HDAC6 was observed in the aorta of TAAD patients, we hypothesize that reduced HDAC6 will affect VSMC function.

Our results showed that decreased HDAC6 resulted in elevated H3K23ac levels which bind to the MMP2, COL1A2, COL3A1, POSTN, ACTA2, and TIMP2 genes to regulate TAAD formation. As a rule, histone acetylation results in an open modification of chromatin structure and facilitates gene transcription (Pons et al., 2009). According to several studies, in the aortas of TAAD patients, type I/III collagen, and CTGF expression are increased. This scenario may be due to an elevation of growth factor or just a slow compensatory process triggered by elastic fiber fragmentation and depletion (Wu et al., 2013; Wang et al., 2006). In addition, matrix metalloproteinase (MMP) dysregulation can lead to ECM destruction in the aortic wall of TAAD patients. Koullias et al. demonstrated that higher MMP2 and MMP9 were observed in the aortas of AD patients than in the control group (Koullias et al., 2004). Surprisingly, inhibitors of metalloproteinases (TIMPs), especially TIMP1 and TIMP2, which were expected to decrease, were upregulated in the dissected aortic wall (Wu et al., 2013; Lesauskaite et al., 2001). Therefore, the imbalance between MMP and TIMP expression may be responsible for the shift towards a proteolytic state in the aorta of AD individuals. Furthermore, previous studies also demonstrated that HDAC6 activity was suppressed by Cathepsin S inhibition in VSMCs in response to PDGF-BB (Wu et al., 2016). Cathepsin K accelerates MMP-2/− 9 expression and activity to control injury-related vascular repair (Hu et al., 2014). However, whether cathepsins regulate several types of cardiovascular diseases, including atherosclerosis, coronary artery disease, and vascular injury (Cheng et al., 2011; Li et al., 2014; Cheng et al., 2012), or play critical roles in AD pathologic processes is unclear.

Histone acetylation always cooperates with histone methylation to regulate chromatin organization and gene expression, and eventually participates in the biological processes of disease. Our recent research findings demonstrated that the histone methyltransferase EZH2 inhibits VSMC autophagic death via blocking autophagosome formation to affect TAAD (Li et al., 2018). Jmjd1a, a histone demethylase, regulates the smooth muscle cell differentiation marker gene (e.g.*,* SM α-actin and SM22) expression by di-methylating H3K9 (H3K9me2) (Lockman et al., 2007). Histone acetylation also plays a critical role in regulating VSMC differentiation (Liu et al., 2015). In this regard, more comprehensive and in-depth research should be performed to unravel the histone PTM landscapes in TAAD patients, which will broaden our understanding of the pathogenesis of TAAD, and provide potential therapeutic targets for TAAD. Our results revealed that the protein levels of H3K9me2, H3K23me1, H3K23ac, and H4K12ac were notably elevated, while H4K20me2, H3K18ac, H4K5ac, and H4K8ac were remarkably reduced in TAAD patients compared with CAD patients. These new findings indicate that histone PTMs, especially acetylation, play an irreplaceable role in human TAAD formation. Furthermore, we identified H3K23ac and H4K20me2 as targets regulated by HDAC6 in TAAD patients.

Multiple signaling pathways, including AKT, MAPK, NF-κB, and TGFβ, participate in regulating VSMC apoptosis, ECM secretion, and inflammation, which are closely related to AD formation (Shen et al., 2013). In our present study, we found that the phosphorylation level of AKT was decreased in the aortic wall of TAAD patients, which was in accordance with published results (Shen et al., 2013). Akt2-deficient mice developed aortic aneurysm/dissection (AAD) after they had been stimulated with angiotensin II, and displayed inflammatory cell infiltration, apoptotic cell death, and tissue destruction in their aortas (Shen et al., 2013). Along with the alteration of signal molecules, the HDAC6 expression level was significantly reduced in the aortas of TAAD patients. Does HDAC6 interact with these signaling pathways in the aorta? Existing research may provide some clues for us to answer this question. Inhibition of HDAC6 led to an increase in AKT activation (p-AKT) to control the survival of tumor cells (Kaliszczak et al., 2016). In neuronal cells, HDAC6 physically interacts with AKT to deacetylate AKT at Lys^163^ and Lys^377^ to regulate the kinase activity of AKT (Iaconelli et al., 2017). In addition to AKT, β-catenin is also a target of HDAC6. In human iPSC-derived neuronal cells, HDAC6 inhibitors facilitate β-catenin acetylation at Lys^49^ and phosphorylation at Ser^45^, and Lys^49^ acetylation of β-catenin results in decreased ubiquitination of β-catenin but increased membrane localization of β-catenin (Iaconelli et al., 2015). In contrast, *Mak* et al. demonstrated that HDAC6 interacts with β-catenin to decrease its acetylation and protect against degradation (Mak et al., 2012). However, our results demonstrated that in VSMCs, the phosphorylation levels of AKT, β-catenin, and AMPKα were enhanced after treatment with the HDAC6 inhibitor tubastatin A. In contrast, MEK1/2 was inactivated by tubastatin A which was consistent with the results observed in TAAD patients, in which decreased HDAC6 was accompanied by reduced MEK1/2 phosphorylation levels. Based on the studies mentioned above and our research findings, we conclude that HDAC6 acts with MEK1/2 signaling to regulate TAAD formation in human beings.

Notably, *Wu* et al. demonstrated that inhibition of Cathepsin S led to a decrease in HDAC6 activity in VSMCs in response to PDGF-BB, and inhibition of HDAC6 by tubastatin A suppressed VSMC proliferation and neointimal hyperplasia in response to injury (Wu et al., 2016). Although we found that the HDAC6 protein levels were remarkably downregulated in the aortic walls of TAAD patients, whether p-HDAC6 and its activity changed during the process of TAAD requires further investigation. The results of Wu’s study indicated that the TLR2-mediated p38MAPK and PI3K-AKT/p-HDAC6 signaling pathways are essential for VSMC migration and proliferation (Wu et al., 2016). Based on these results, we conducted an in-depth study and demonstrated that HDAC6 was involved in human TAAD formation by regulating H3K23ac, H4K20me2 and p-MEK1/2. Additionally, HDAC6 is a sex-related member of the HDAC family. According to the International Registry of Aortic Dissection (IRAD) registry, 65% of aortic dissection patients were men (Erbel et al., 2014). Therefore, in our current study, we did not evaluate the differences in the levels of HDAC6 and p-HADAC6 proteins between men and women. This is another limitation of our present study.

## Conclusions

We report that H3K9me2, H3K23me1, H4K12ac, and H3K23ac levels increased, while H4K20me2, H3K18ac, H4K8ac, and H4K5ac levels are reduced in the aortas of TAAD patients compared with CAD patients. Furthermore, we found that HDAC6 deacetylates H3K23 to regulate the ECM arrangement of the aorta. Based on the results that HDAC6, H4K20me2 and p-MEK1/2 were decreased in TAAD patients and tubastatin A treatment inhibited H4K20me2 and p-MEK1/2 levels, we concluded that HDAC6 was involved in TAAD formation via regulating H3K23ac, H4K20me2, and p-MEK1/2.

## Additional file

Additional file 1: **Table S1.** Primers for ChIP-PCR of H3K23ac. (DOCX 14 kb)

## Abbreviations

AD: Aortic dissection
CAD: Coronary artery disease
ChIP-PCR: Chromatin immunoprecipitation- polymerase chain reaction
ECM: Extracellular matrix
ECs: endothelial cells
HDAC: Histone deacetylase
MEF2: Myocyte enhancer factor-2
MMPs: Matrix metalloproteinases
PAH: Pulmonary arterial hypertension
PASMCs: pulmonary artery smooth muscle cells
PTMs: Post-translational modifications
SD: Standard deviation
TAAD: Type A aortic dissection
TIMPs: inhibitor of metalloproteinases
VSMCs: Vascular smooth muscle cells
