# Supplementary material for: HDAC6 is associated with the formation of aortic dissection in human
Source: Mol Med. 2019 Mar 29;25:10. doi: 10.1186/s10020-019-0080-7 (PMC6441237; doi:10.1186/s10020-019-0080-7)
Supplement: Supplementary file 1 — Table S1. Primers for ChIP-PCR of H3K23ac. (DOCX 14 kb) [file 10020_2019_80_MOESM1_ESM.docx]

**Supplemental materials**

**Table S1. Primers for ChIP-PCR of H3K23ac.**

| Gene Name |  | Primers |
| --- | --- | --- |
| ACTA2 | Forward | CCGGGACTAAGACGGGGTAA |
|  | Reverse | GCATCTGGACCCTCCTACCT |
| TIMP2 | Forward | TTTGCTAAAGGGAGGTGCCC |
|  | Reverse | GTGGGACTTTGGCCTCTCTC |
| MMP2 | Forward | CCTACCCCTGTGCTTTGGAA |
|  | Reverse | AACCACAACTCCTTGCCACA |
| COL3A1 | Forward | CTACTATCCCCCACCAACCA |
|  | Reverse | CTTGCAACCACGAGGCAAC |
| COL1A2 | Forward | GTAGGGAGTGGAGGGTTGGA |
|  | Reverse | ATTCGAGCTTGGGGTCTTCG |
| CTGF | Forward | ATTGATGGCCACTCCTCCCT |
|  | Reverse | AGTCTCCTGGGGCAGATTTC |
| POSTN | Forward | CAGTCCTTTGCCTGCCTCTAA |
|  | Reverse | ACAGCAGAGAGTAGTGTCCCT |
